# Sentinel serosurveillance of backyard hens proved West Nile virus circulation in the western provinces of Turkey

**DOI:** 10.1002/vms3.589

**Published:** 2021-07-29

**Authors:** Harun Albayrak, Ismail Sahindokuyucu, Bahadir Muftuoglu, Cuneyt Tamer, Hamza Kadi, Emre Ozan, Ozge Yilmaz, Hamza Kilic, Hanne Nur Kurucay, Fethiye Coven, Semra Gumusova, Zafer Yazici, Ahmed Eisa Elhag

**Affiliations:** ^1^ Department of Virology, Faculty of Veterinary Medicine Ondokuz Mayis University Samsun Turkey; ^2^ Bornova Veterinary Control Institute Ministry of Agriculture and Forestry Izmir Turkey; ^3^ Department of Experimental Animals, Faculty of Veterinary Medicine Ondokuz Mayis University Samsun Turkey; ^4^ Samsun Veterinary Control Institute Ministry of Agriculture and Forestry Samsun Turkey; ^5^ Department of Preventive Medicine and Clinical Studies, Faculty of Veterinary Sciences University of Gadarif Al Qadarif Sudan

**Keywords:** backyard hens, ELISA, seropositive, Turkey, West Nile virus

## Abstract

West Nile virus (WNV) is a mosquito‐borne virus of a re‐emergence importance with a wide range of vertebrate hosts. Granted, it causes asymptomatic infection, but fatal cases and neurologic disorders were also recorded, especially in humans, horses and some exposed birds. The virus is globally spread and birds are considered an amplifying and reservoir host of WNV, helping to spread the disease due to their close contact with main hosts. In this study, we aimed to detect the presence of antibodies against WNV in backyard hens that were reared in the western Anatolian part of Turkey. A total of 480 chicken sera were randomly collected from six provinces in the west of Turkey (Mugla, Izmir, Aydin, Afyonkarahisar, Kutahya and Manisa) with 80 samples from each province (40 in spring and 40 in fall seasons). They were tested by using a competitive ELISA method to identify the specific avian antibodies of IgG that produced against the WNV envelope proteins (pr‐E). Twelve of 480 (2.5%) sera were found seropositive, three of these positive sera were detected from the Izmir province (3.75%) collected in the spring session and the other nine positive sera were detected from the Mugla province (11.25%) collected in the fall session. Both of these provinces are located seaside and have suitable climate conditions for vectors of infection. The results indicated that WNV infection is in circulation in these provinces, and that may put the other susceptible vertebrates under risk of infection.

## Graphical abstract

Our study is considered for notifying serologically the presence of antibodies against West Nile virus (WNV) in backyard hens that reared in the western part of Turkey, to see if it can pose a substantial risk for both public and animal health in these areas, and the obtained results confirmed this hypothesis by achieving a seroprevalence rate of 2.5%. This outcome has an imperative significance for enhancing early detection sensitivity and capacity tools that could be used for indicating WNV circulation and estimating a possible future occurrence of human cases because those local chickens that we used in our study can play the role of sentinel animals that can provide early detection of WNV transmission.

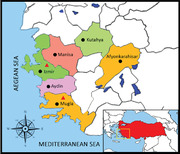

## 1 INTRODUCTION

West Nile virus (WNV) is classified under the *Flavivirus* genus of *Flaviviridae* family and is known as a member of the Serocomplex Group of Japanese Encephalitis (JE) virus, which also includes three other viruses: Kunjin (KUN), Murray Valley encephalitis (MVE), and St Louis Encephalitis (SLE) viruses (Yazici et al., 2012). Also, (WNV) is an “old‐world” enveloped arthropod‐borne virus (Arbovirus), transmitted mostly by carrier‐mosquitoes and has a single‐stranded RNA genome weighing 11 kb with positive sense polarity. The virus was isolated for the first time from a human case in 1937 in Uganda and future cases were reported in many places such as Asia, Africa, parts of Europe, Australia (Kunjin virus) and North America as an endemic disease (Kecskeméti et al., 2013). WNV is one of the important zoonotic viruses which causes severe neurological disease with serious symptoms including high fever, encephalitis and death. The disease is seen in many mammals, but the most susceptible are humans and horses, hosts considered to be for the virus transmission cycle as dead‐end because their contribution to it is ineffective (Madic et al., 2013; Ozan et al., 2019). A diagnosis of the WNV disease is generally accomplished by using serological methods. Whilst for a specific diagnosis, the gold standard is thought to be the plaque reduction neutralization test. ELISA presently is utilized ordinarily, as it is not so much relentless but rather more fit to high throughput screening (Castillo‐Olivares & Wood, 2004; Pir & Albayrak, 2017). While WNV is a zoonotic infection, horses are more inclined to WNV than humans and 10% of infected steeds may develop neurological symptoms (Castillo‐Olivares & Wood, 2004). Currently, there is no accessibility for vaccination or treatment for humans, in spite of commercially available inactivated and recombinant vaccines for steeds (Seino et al., 2007).

Many species of birds do not develop any WNV disease, so they play a crucial role as an amplifier and reservoir host in virus circulation, especially the wild ones. However, several species such as crows, jays and hawks may die of WNV infection (Gamino & Hofle, 2013). On the other hand, various species (mammals to include humans and horses, amphibians and domestic birds) could acquire the infection by WNV contaminated mosquito bites (essentially by *Culex* spp). Additionally, ticks and other arthropods can also play a role in the viral transmission (Albayrak & Ozan, 2010; Albayrak & Ozan, 2013; Jiménez de Oya, et al., 2019; Liang et al., 2015). There were many reports about the serological and virological evidence of WNV infection in humans, animals and birds in Turkey, which is a major Eurasian crossroad for movement of people and birds (Albayrak & Ozan, 2013; Gazi et al., 2016; Pir & Albayrak, 2017; Toplu et al., 2015). All WNV cases of humans were identified exclusively in the west side of Turkey at the Aegean region. This region, which is where this study was conducted, is more suitable as a natural and ecological condition for mosquitoes than other parts of Turkey. It is also a border between Turkey and Greece, wherein multiple WNV human cases were observed in 2010, with 18 human fatalities (Kalaycioglu et al., 2012).

Therefore, it is very important to screen the WNV infection in bird populations occupying that part of Turkey, which is considered to be favourable for the virus vectors (*Culex* spp) (Albayrak & Ozan, 2013). According to the way of virus transmission and gabs that related to relevant vaccination and treatment protocols, it is obvious that a collaboration strategy is needed between many national and international authorities that work in animal and public health sectors to support all efforts forwarded against WNV surveillance and control programs, particularly the focus on arthropods and avians within the One Health initiative. Bearing in mind the presence of sentinel hosts that give the most advantageous source of blood for observing WNV transmission, and in such cases, both captive and backyard chickens play a big role in this matter as being the favored blood‐feeding hosts for the main WNV vector (*Culex* spp) in Turkey's surrounding countries (Petric et al., 2012). All aforementioned data encouraged us to run a serological survey to investigate the presence of antibodies against WNV in backyard hens reared in the western part of Turkey, to see if they may pose a substantial risk for both public and animal health in western Anatolia.

## 2 MATERIALS AND METHODS

### 2.1 Collection and processing of samples

**FIGURE 1:**
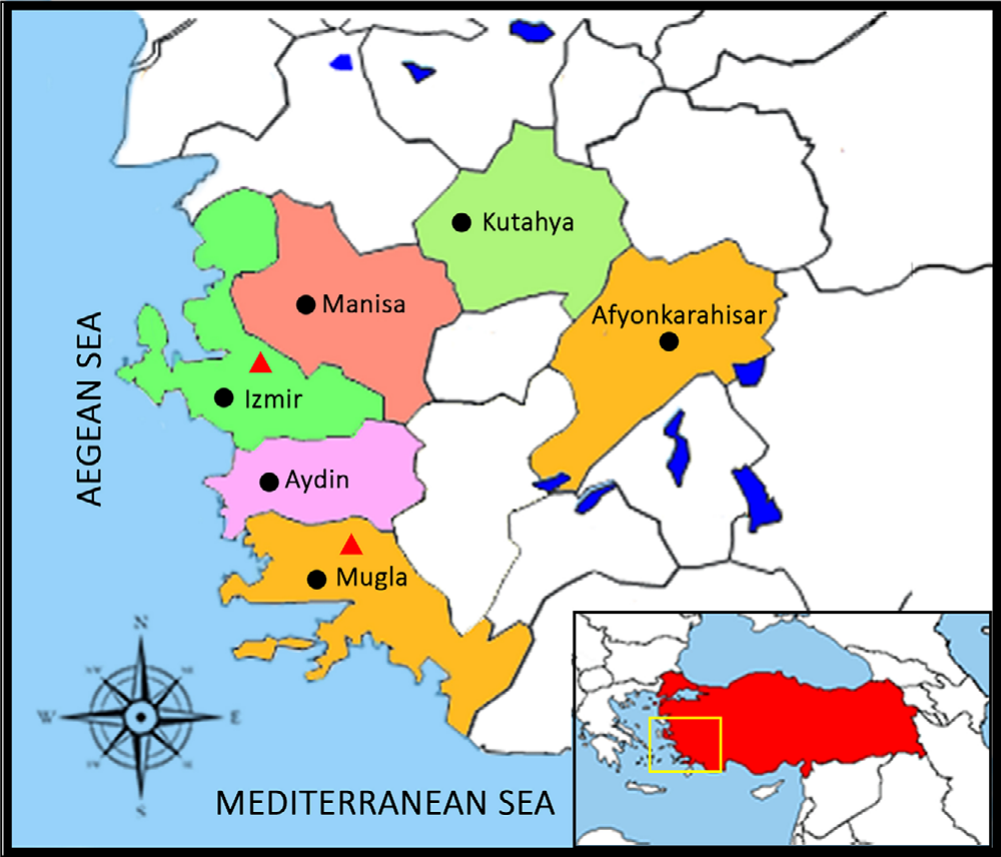
Map of Turkish provinces where hens sera were collected. ^●^Provinces where serum was collected. ^▲^Provinces where positive serum was found

The present study was performed by using 480 Archival blood samples that collected in the spring (240) and the fall (240) of 2018 from randomly selected backyard hens located in six different provinces (Mugla, Izmir, Aydin, Afyonkarahisar, Kutahya and Manisa) of the Western Anatolia region with 80 samples from each province (40 in spring and 40 in fall seasons), this region geographically lies in the west of Turkey as depicted in Figure 1. The sample size and random sampling procedure were determined by using the described method of Houe et al. (2004), in which a selection was made without regard to exposure or disease status.

After taking blood samples from the hens wing veins (<1 year), collected from each province, it was then transported to the laboratory under the cold chain. Blood samples were centrifuged at 1500 rpm for 10 min. After that serum samples were aliquoted into sterile tubes then inactivated for 30 min at 56°C and stored until investigation at –20°C.

### 2.2 Competitive ELISA for WNV

Samples were tested using commercially available enzyme‐linked immunosorbent assay (IgG ELISA) kits produced by (ID.Vet, Montpellier, France) for the detection of avian antibodies against the West Nile virus, and the test was performed and interpreted following manufacturer's instructions. The designation of this diagnostic kit is based on the detection of IgG antibodies that point towards the WNV envelope protein (pr‐E) by competitive ELISA.

This assay is used for identifying anti‐WNV immunoglobulins, and their presence is interpreted as a positive result. All samples were examined twice and reading of plates by ELISA reader at 450 nm and the calculation of results was done as well.

The threshold value for considering a serum as positive was %OD sample/negative control (S/N). We calculated residual binding rations (S/N%) in order to obtain The ELISA validation. Any detected S/N ratios equal to or less than 40% in the serum samples have been considered as positive; while negative samples accepted when it has above 50% ratios. As recommended by the manufacturer the doubtful results were considered when S/N values ranged between 40% and 50%.

## 3 RESULTS

As detailed in Table 1, a total of 480 serum samples collected from backyard chickens were tested by C‐ELISA to detect IgG antibodies against WNV. An overall seroprevalence for WNV was found to be 2.5% (12/480). Three of these positive sera were detected from the Izmir province (3.75%) collected in the spring session and the other nine positive sera were detected from the Mugla province (11.25%) collected in the fall session.

**TABLE 1:** The number and ratio of WNV seropositive backyard hens

Province	Total number of chickens	Positivity (%) (for WNV)
Mugla	80	9 (11.25%)
Izmir	80	3 (3.75%)
Aydin	80	0
Afyonkarahisar	80	0
Kutahya	80	0
Manisa	80	0
Total	480	12 (2.5%)

In all backyard chickens that tested from the Aydın, Afyonkarahisar, Kutahya and Manisa provinces no anti‐WNV antibodies were detected. The highest provincial distribution rate of WNV seropositivity (11.25%) was detected in the Mugla province, and the second seropositivity rate (3.75%) was detected in the Izmir province. Both of these provinces are located seaside and have suitable climate conditions for vectors of infection.

## 4 DISCUSSION

The normal transmission cycle of WNV is between its arthropod vector and birds. Through this mosquito‐bird cycle, which involves primarily, ornithophilic mosquitoes belonging to the *Culex* species, the virus is able to be maintained and spread. However, the virus could reach a variety of incidental vertebrate hosts like horses and humans, through transmission by anthropophilic and/or mammophilic mosquito species (*Ochlerotatus spp*. and *Aedes spp*.), resulting usually in asymptomatic infection, but it can lead to meningoencephalitis in horses and humans (Colpitts et al., 2012; Yazici et al., 2018). As transmission is directly related to environmental conditions, flood plains, river deltas and the migratory birds routes together with many other factors such as humidity, wetlands ecosystems, an abundance of avifauna and mosquitoes, and so on can strongly affect the spread of WNV and will constitute an appropriate foci of its infections (Lafri et al., 2017). All aforementioned ecological and climatic conditions suitable for WNV are available in Turkey, especially in the provinces of our target study which resided northeast of the Mediterranean basin, potentially considered an endemic area for harbouring WNV and many arboviral infections, because the disease was reported in many birds, mammalian species and mosquitoes, besides the presence of confirmed human and horses outbreaks in that area of Turkey (Ozer et al., 2007). On top of that pieces of evidence for the presence of WNV in Greece and Bulgaria were confirmed. Therefore, these are also neighbouring countries for the target regions of our study (Calistri et al., 2010). On the other shore of the Aegean and the Mediterranean Sea, especially in the central, southern and western parts of Turkey, the presence of WNV was reported for the first time serologically in different animal species including horses (13.5%), ass‐mules (2.5%), dogs (37.7%), cattle (4%), sheep (1%) and also in humans (20.4%) (Ozkul et al., 2006).

There are not many survey studies concentrated on birds of Turkey, neglecting that the key factor for transmission of WNV in birds due to their natural rule in virus amplification. In addition, the virus has been isolated from various wetland and terrestrial avian species in different areas (Hamer et al., 2009; McLean et al., 2002). WNV did not affect turkeys (*Meleagridis gallopavo*) or commercial chickens (*Gallusgallus domesticus*), which have dominatingly low mosquito‐vectors exposure potentiality due to their indoor raising (Komar et al., 2003). Yet, an infection of WNV that was associated with severe neurologic symptoms and death occurred naturally and was recorded in old domestic geese from a flock in Israel (Malkinson et al., 2001). In this Israeli outbreak, the goose role as a reservoir for WNV is unknown, however, 27% were recorded as WNV infection rates in other groups of geese belonging to the District of Sindbis located off the northern Nile Valley, which may suggest a leading role goose could play in the local ecology patterns of WNV (Swayne et al., 2001). A proof of WNV transmission was given ahead of time, earlier than USA human cases by sentinel chicken‐based WNV surveillance attempts (Healy et al., 2012). In spite of the high susceptibility of horses towards the infection, however, the possibility of their use as sentinel animals cannot be achieved in places where WNV vaccination of its population is thoroughly used. Nevertheless, we decided to include backyard hens (instead of farmed and captive hens) as sentinel animals in our survey, as it is less economically demanding, and they respond more precisely positive for serosurveys according to prior studies. The suitability of backyard hens for early estimation of WNV circulation was demonstrated in numerous countries such as Greece (Chaintoutis et al., 2016). All aforementioned data were effectively hired for discovering the early circulation of the virus before the starting of any human cases, which could be utilized by the public health authorities who work locally and internationally as a subsequent rapid notification system (Chaintoutis et al., 2016). This encouraged us to employ the C‐ELISA technique in this study which, for WNV antibodies, has a higher sensitivity (84.9%) and specificity (99.4%) (Pir & Albayrak, 2017), to determine the WNV seropositivity in backyard hens of western Turkey, which was found to be 2.5%. This percentage, which is close to many founded WNV prevalence rates in our country, like Pir and Albayrak (Pir & Albayrak, 2017), who performed a serological survey in the northern part of Turkey in some domestic birds like (duck, turkey, goose and chicken), found 32 of 736 collected sera were positive (4.3%). The distribution of their seropositivity rates was chicken 3.1%, duck 0.8%, goose 1.8% and turkey 17.9%. Also in the same region, Albayrak and Ozan (Albayrak & Ozan, 2010) implemented a molecular study on WNV infection in wild birds, but they did not find any WNV nucleic acid from the collected swab and organ samples.

Our present findings and the results of surveys and studies that had been carried previously during the 2010 epidemic of Greece, highlight the urgent importance of constructing a model system like sentinel hens that are able to predict the dynamics of WNV in certain areas and to give reliable outcomes that can help in determining minimum and earlier activity of the virus, along with the overall influence of viral spreading in the absence of human cases, because the immunity of these crucial birds hosts is assumed to play a significant role in identifying viral amplification and transmission to human (Kwan et al., 2012). Contrarily, the observation for distinguishing proofs to low‐level arboviruses transmission in explicit geological regions by utilizing sentinels, does not really suggest that clinical manifestations will occur in humans later (Chaintoutis et al., 2016). The results of our study support previous studies and clarify the value of domestic avian‐based WNV surveillance for observing any muted amplification of the virus. But in order to quantify and further understand those values, all influences related to the spreading of arboviruses should be co‐analyzed.

We found positive relationships between temperature anomalies and detection of WNV, as our prevalence rates were 0.6% for the spring season and 1.9% for the fall season. These speculations could be a result of temperature effects, especially in the spring and at the start of fall heat, based on the gonotrophic pattern of the mosquito vector. The extraneous incubation period of WNV was in accordance with previous studies that distinguished positive connections between WNV risk and temperature (Morin & Comrie, 2013). An increase in temperature has been proved to have an effect on raising the intensity of WNV transmission (Chuang & Wimberly, 2012). We detected a higher positive value at the beginning of the fall season than the spring, in which temperature anomalies and humidity may take part in increasing host‐seeking activity of mosquitoes which can encourage the continued amplification of virus as well as the rise of infected mosquitoes that bite avians with higher rates, in particular, backyard hens (Kilpatrick et al., 2006).

## 5 CONCLUSION

This study revealed WNV infection is in circulation in the western part of Turkey, which means people who live in those areas are under the risk of WNV infection, because backyard hens may be considered sentinel animals for detecting WNV transmission. Based on the obtained results and foreseen continued extreme flow of WNV, further epidemiological research beside continuity of WNV infection surveillance and monitoring programs for coming years in Turkey will be of imperative significance to improve the sensitivity to make a quick diagnosis and to enhance the capability and capacity for indicating WNV circulation and estimating a possible future occurrence of human cases.

## ETHICS STATEMENT

The authors confirm that the ethical policies of the journal, as noted on the journal's author guidelines page, have been adhered to. Even though archived samples were used, we conducted this study under ethical standards approved by the Samsun Veterinary Control Institute Scientific Ethics Committee, Ministry of Agriculture and Forestry, Republic of Turkey (No: 19972899/03/‐52, Date: 05/07/2019), also legal permission was taken from Ministry of Agriculture and Forestry, Republic of Turkey (No: 71037622‐604‐02‐E.2823063, Date: 17/09/2019), because WNV infection is accepted as a notifiable disease in Turkey.

## AUTHOR CONTRIBUTIONS

Harun Albayrak was associated with conceptualization and supervision. Ismail Sahindokuyucu was associated with formal analysis, investigation and resources. Bahadir Muftuoglu was associated with methodology, software and wrote the original draft. Cuneyt Tamer was associated with formal analysis and methodology. Hamza Kadi was associated investigation and resources. Emre Ozan was associated with validation and visualization. Ozge Yilmaz was associated with investigation and resources. Hamza Kilic was associated with investigation and resources. Hanne Nur Kurucay was associated with methodology. Fethiye Coven acquired the resources. Semra Okur‐Gumusova was associated with conceptualization. Zafer Yazici was associated with conceptualization, supervision, visualization and reviewed and edited the final manuscript. Ahmed Eisa Elhag was associated with formal analysis, methodology and reviewed and edited the final manuscript.

## CONFLICT OF INTEREST

The authors declare that there is no conflict of interest.

### PEER REVIEW

The peer review history for this article is available at https://publons.com/publon/10.1002/vms3.589.

## References

[vms3589-bib-0001] Albayrak, H. , & Ozan, E. (2010). Molecular detection of avian influenza virus but not West Nile virus in wild birds in northern Turkey. Zoonoses Public Health, 57, 71–75. 20298488 10.1111/j.1863-2378.2010.01327.x

[vms3589-bib-0002] Albayrak, H. , & Ozan, E. (2013). Seroepidemiological study of West Nile virus and rift valley Fever virus in some of Mammalian species (herbivores) in northern Turkey. Journal of Arthropode Borne Diseases, 7(1), 90–93. PMC3684502 23785699

[vms3589-bib-0003] Calistri, P. , Giovannini, A. , Hubalek, Z. , Ionescu, A. , Monaco, F. , Savini, G. , & Lelli, R. (2010). Epidemiology of west nile virus in Europe and in the Mediterranean basin. Open Virology Journal, 4, 29–37. 10.2174/1874357901004010029 PMC2878979 20517490

[vms3589-bib-0004] Castillo‐Olivares, J. , & Wood, J. (2004). West Nile virus infection of horses. Veterinary Research, 35, 467–483. 15236677 10.1051/vetres:2004022

[vms3589-bib-0005] Chaintoutis, S. C. , Gewehr, S. , Mourelatos, S. , & Dovas, C. I. (2016). Serological monitoring of backyard chickens in Central Macedonia‐Greece can detect low transmission of West Nile virus in the absence of human neuroinvasive disease cases. Acta Tropica, 163, 26–31. 27469618 10.1016/j.actatropica.2016.07.018

[vms3589-bib-0006] Chuang, T. W. , & Wimberly, M. C. (2012). Remote sensing of climatic anomalies and West Nile virus incidence in the northern Great Plains of the United States. PLoS ONE, 7, e46882. 23071656 10.1371/journal.pone.0046882 PMC3465277

[vms3589-bib-0007] Colpitts, T. M. , Conway, M. J. , Montgomery, R. R. , & Fikrig, E. (2012). West Nile virus: Biology, transmission, and human infection. Clinical Microbiology Reviews, 25, 635–648. 23034323 10.1128/CMR.00045-12 PMC3485754

[vms3589-bib-0008] Gamino, V. , & Hofle, U. (2013). Pathology and tissue tropism of natural West Nile virus infection in birds: A review. Veterinary Research, 44, 39. 23731695 10.1186/1297-9716-44-39 PMC3686667

[vms3589-bib-0009] Gazi, H. , Özkütük, N. , Ecemis, T. , Atasoylu, G. , Köroglu, G. , Kurutepe, S. , & Horasan, G. D. (2016). Seroprevalence of west nile virus, crimean‐congo hemorrhagic fever virus, Francisella tularensis and Borrelia burgdorferi in rural population of Manisa, western Turkey. Journal of Vector Borne Diseases, 53, 112–117. 27353580

[vms3589-bib-0010] Hamer, G. L. , Kitron, U. D. , Goldberg, T. L. , Brawn, J. D. , Loss, S. R ,, Ruiz, M. O. , Hayes, D. B. , & Walker, E. D. (2009). Host selection by Culex pipiens mosquitoes and West Nile virus amplification. American Journal of Tropical Medicine and Hygiene, 80, 268–278. 19190226

[vms3589-bib-0011] Healy, J. , Reisen, W. K. , Kramer, V. , & Barker, C. M. (2012). Do current surveillance methods provide adequate warning for human infections with West Nile virus? Proc. Mosq. Control Assoc. Calif., 80, 17–21.

[vms3589-bib-0012] Houe, H. , Ersboll, A. K. , & N. Toft (Eds.) (2004). Introduction to Veterinary Epidemiology. 1st edn. Multiple testing: Chapter 9.6. Biofolia. pp. 147–148. Narayana Press, Gylling, Denmark.

[vms3589-bib-0013] Jiménez de Oya, N. , Escribano‐Romero, E. , Camacho, M. C. , Blazquez, A. B. , Martín‐Acebes, M. A. , Höfle, U. , & Saiz, J. C. (2019). A Recombinant subviral particle‐based vaccine protects magpie (Pica pica) against West Nile virus infection. Frontiers in Microbiology, 10, 1133. 31231320 10.3389/fmicb.2019.01133 PMC6560071

[vms3589-bib-0014] Kalaycioglu, H. , Korukluoglu, G. , Ozkul, A. , Oncul, O. , Tosun, S. , Karabay, O. , Gozalan, A. , Uyar, Y. , Caglayık, D. Y. , Atasoylu, G. , & Altas, A. B. (2012). Emergence of West Nile virus infections in humans in Turkey, 2010 to 2011. Euro Surveillance, 17(21), 20182. 22687827

[vms3589-bib-0015] Kecskeméti, S. , Bajmócy, E. , Bacsadi, Á. , Kiss, I. , & Bakonyi, T. (2013). Encephalitis due to West Nile virus in a sheep. Veterinary Record, 161, 568–569. 10.1136/vr.161.16.568 17951568

[vms3589-bib-0016] Kilpatrick, A. M. , Kramer, L. D. , Jones, M. J. , Marra, P. P. , & Daszak, P. (2006). West Nile virus epidemics in North America are driven by shifts in mosquito feeding behavior. PLoS Biology, 4, e82. 16494532 10.1371/journal.pbio.0040082 PMC1382011

[vms3589-bib-0017] Komar, N. , Langevin, S. , Hinten, S. , Nemeth, N. , Edwards, E. , Hettler, D. , Davis, B. , Bowen, R. , & Bunning, M. (2003). Experimental infection of North American birds with the New York 1999 strain of West Nile virus. Emerging Infectious Diseases, 9(3), 311–322. 12643825 10.3201/eid0903.020628 PMC2958552

[vms3589-bib-0018] Kwan, J. L. , Kluh, S. , & Reisen, W. K. (2012). Antecedent avian immunity limits tangential transmission of West Nile virus to humans. PLoS ONE, 7, e34127. 22457819 10.1371/journal.pone.0034127 PMC3311586

[vms3589-bib-0019] Lafri, I. , Prat, C. M. , Bitam, I. , Gravier, P. , Besbaci, M. , Zeroual, F. , Ben‐Mahdi, M. H. , Davoust, B. , & Leparc‐Goffart, I. (2017). Seroprevalence of West Nile virus antibodies in equids in the North‐East of Algeria and detection of virus circulation in 2014. Comparative Immunology Microbiology and Infectious Diseases, 50, 8–12. 10.1016/j.cimid.2016.11.005 28131384

[vms3589-bib-0020] Liang, G. , Gao, X. , & Gould, E. A. (2015). Factors responsible for the emergence of arboviruses; strategies, challengers and limitations for their control. Emerging Microbes and Infections, 4(3), e18. 26038768 10.1038/emi.2015.18 PMC4395659

[vms3589-bib-0021] Madic, J. , Savini, G. , Di Gennaro, A. , Monaco, F. , Jukic, B. , Kovac, S. , Rudan, N. , & Listes, E. (2013). Serological evidence for West Nile virus infection in horses in Croatia. Vetetrinary Record, 160, 772–773. 10.1136/vr.160.22.772 17545649

[vms3589-bib-0022] Malkinson, M. , Banet, C. , Khinich, Y. , Samina, I. , Pokamunski, S. , & Weisman, Y. (2001). Use of live and inactivated vaccines in the control of West Nile fever in domestic geese. Annals of the New York Academy of Sciences, 951, 255–261. 11797782 10.1111/j.1749-6632.2001.tb02701.x

[vms3589-bib-0023] McLean, R. G. , Ubico, S. R. , Bourne, D. , & Komar, N. (2002). West Nile virus in livestock and wildlife. Current Topics in Microbiology and Immunology, 267, 271–308. 12082994 10.1007/978-3-642-59403-8_14

[vms3589-bib-0024] Morin, C. W. , & Comrie, A. C. (2013). Regional and seasonal response of a West Nile virus vector to climate change. Proceedings of the National Academy of Sciences of USA, 110(39), 15620–15625. 10.1073/pnas.1307135110 PMC3785720 24019459

[vms3589-bib-0025] Ozan, E. , Albayrak, H. , Gumusova, S. , Bolukbas, C. S. , Kurt, M ,, Pekmezci, G. Z. , Beyhan, Y. E. , Kadi, H. , Kaya, S. , Aydin, I. , & Yazici, Z. (2019). A study on the identification of five arboviruses from hematophagous mosquitoes and midges captured in some parts of Northern Turkey. Journal of Arthropod Borne Diseases, 13(2), 224–233. 31803784 PMC6885145

[vms3589-bib-0026] Ozer, N. , Ergunay, K. , Simsek, F. , Kaynas, S. , Alten, B. , Caglar S. S. , & Ustacelebi S. (2007). West Nile virus studies in the Sanliurfa region province of Turkey. Journal of Vector Ecolology, 32, 202–206. 10.3376/1081-1710(2007)32[202:wnvsit]2.0.co;2 18260509

[vms3589-bib-0027] Ozkul, A. , Yıldırım, Y. , Pinar, D. , Akcali, A. , Yilmaz, V. , & Colak D. (2006). Serological evidence of West Nile virus (WNV) in mammalian species in Turkey. Epidemiology and Infection, 134, 826–829. 16316496 10.1017/S0950268805005492 PMC2870448

[vms3589-bib-0028] Petric, D. , Hrnjakovic‐Cvjetkovic, I. , Radovanov, J. , Cvjetkovic, D. , Jerant‐Patic, V. , Milosevic, V. , Kovacevic, G. , Zgomba, M. , Ignjatovic‐Cupina, A. , Konjevic, A. , & Marinkovic, D. (2012). West Nile virus surveillance in humans and mosquitoes and detection of cell fusing agent virus in Vojvodina province (Serbia). HealthMED, 6(2), 462–6.

[vms3589-bib-0029] Pir, S. , & Albayrak, H. (2017). Serological evidences of West Nile virus in domestic bird species in the Samsun province. Etlik Veteriner Mikrobiyoloji Dergisi, 28, 105–108.

[vms3589-bib-0030] Seino, K. K. , Long, M. T. , Gibbs, E. P. , Bowen, R. A. , Beachboard, S. E. , Humphrey, P. P. , Dixon, M. A. , & Bourgeois, M. A. (2007) Comparative efficacies of three commercially available vaccines against West Nile Virus (WNV) in a short‐duration challenge trial involving an equine WNV encephalitis model. Clinical and Vaccine Immunology, 14, 1465–1471. 17687109 10.1128/CVI.00249-07 PMC2168174

[vms3589-bib-0031] Swayne, D. E. , Beck, J. R. , Smith, C. S. , Shieh, W. J. , & Zaki, S. R. (2001). Fatal encephalitis and myocarditis in young domestic geese (Anser anser domesticus) caused by West Nile virus. Emerging Infectious Diseases, 7, 751–753. 11585545 10.3201/eid0704.010429 PMC2631765

[vms3589-bib-0032] Toplu, N. , Oğuzoğlu, T. Ç. , Ural, K. , Albayrak, H. , Ozan, E. , Ertürk, A. , & Epikmen, E. T. (2015). West Nile virus infection in horses: Detection by immunohistochemistry, in situ hybridization, and ELISA. Vetetrinary Pathology, 52, 1073–1076. 10.1177/0300985815570067 25677341

[vms3589-bib-0033] Yazici, Z. , Albayrak, H. , Ozan, E. , & Gumusova, S. (2012). The first investigation of West Nile virus in horses using real‐time RT‐PCR in middle black sea region in Turkey. Journal of Arthropod Borne Diseases, 6(2), 151–155. 23378973 PMC3547302

[vms3589-bib-0034] Yazici, Z. , Tamer, C. , Gumusova, S. , Ozan, E. , Hacioğlu, S. , Ozkul, A. , & Albayrak, H. (2018). Serological signs of West Nile virus infection in horse serum samples collected for Equine Infectious Anemia virus screening in the Northeastern Turkey: Traces of past. Pakistan Veterinary Journal, 39, 1–6.

